# Subjective assessment reported by patients shows differences between single-bundle and double-bundle anterior cruciate ligament reconstruction, systematic review and meta-analysis

**DOI:** 10.1038/s41598-021-94868-0

**Published:** 2021-07-28

**Authors:** Antonio Maestro, Irene Herruzo, David Varillas-Delgado, Carlos Martín-Saborido

**Affiliations:** 1 Begoña Hospital, Gijón, Spain; 2 Faculty of Medicine, Universidad de Oviedo, Oviedo, Spain; 3 Faculty of Medicine, Universidad Francisco de Vitoria, Pozuelo de Alarcon, Madrid, Spain; 4 Faculty of Medicine, Universidad Francisco de Vitoria, Pozuelo de Alarcon, Madrid, Spain; 5 Centro de educación superior Hygiea, UDIMA, Madrid, Spain

**Keywords:** Physiology, Anatomy

## Abstract

To determine the functional recovery, active reincorporation, and anteroposterior and rotational stability of patients undergoing anterior cruciate ligament (ACL) reconstruction using arthroscopy techniques with simple-bundle (SB) or double-bundle (DB). The following databases were searched: PubMed, Embase (Elsevier platform), the Cochrane Central Register of Controlled Trials (Wiley platform), Web of Science, and CINAHL. Level I and II studies involving anterior cruciate ligament arthroscopy were included in the search. Records were screened by title and abstract and assessed the risk of bias of selected studies. Meta-analyses using RevMan 5.3 software were conducted on the following outcomes: knee functionality, objective measurements of knee stability, rotational knee stability and knee anterior stability, sports reincorporation, and subjective assessments. Twenty-four studies of patients undergoing ACL reconstruction were included in the qualitative and quantitative synthesis (1707 patients) for Lysholm score, Subjective International Knee Documentation Committee (IKDC) score, Tegner score, KT-1000/2000, Lachman test, Objective IKDC score, and Pivot-Shift test. A return to pre-injury level showed a significant decrease in the Lysholm score (mean difference, − 0.99; 95% CI − 1.71 to − 0.40; *P* = 0.007) and Tegner score (mean difference, − 0.07; 95% CI, − 0.13 to − 0.01; *P* = 0.02) at DB reconstruction, similar to the knee functionality outcome of the subjective IKDC score (mean difference − 1.42; 95% CI − 2.46 to − 0.38; *P* = 0.007). There is no clear or significant difference in clinical stability and knee function or in sports incorporation with the true difference occurring in the subjective assessment.

## Introduction

Rupture of the anterior cruciate ligament (ACL) represent 50% of ligament injuries of the knee^1,2^. Seventy-five percent of these ruptures occur during sports activities such as football^3,4^, basketball^5^ or skiing^6^. In addition, the prevalence has increased in the latest trends due to increased activity of the population (as high as 3/10.000 individuals/year^7^), which implies a high cost in public health.

The arthroscopic single-bundle (SB) technique is the most common method used in ACL reconstruction^8–10^. This reconstruction technique may provide good clinical outcomes and restore anterior stability following an ACL injury^11^, improve joint stability, proprioceptive function, and balance ability^12^, but it may also be suboptimal concerning rotational function^13^. The arthroscopic double-bundle (DB) strategy, which was first described by Mott in 1983^14^, technically reconstructs 2 functional bundles of the ACL and thereby more closely approximates the native anatomy. Moreover, it demonstrates less anterior laxity by using a KT-1000 arthrometer^15^ and increased objective tibial stability and objective IKDC scores compared to SB ACL reconstruction^16^.

An increasing number of studies and systematic reviews have compared the two surgical techniques, that is, SB versus DB procedures^17–19^. Several clinical studies have reported that anatomic DB ACL reconstruction might increase rotational and anterior stability of the knee^20^, improve graft-tunnel healing^21^ and decrease the rate of meniscal tears^22,23^. Several studies found no significant differences between clinical outcomes in either group with a long follow-up^24–26^. Several meta-analyses have also been published comparing the two procedures (SB vs. DB) and it remains unclear which one is superior in clinical outcomes. Moreover, this was determined when randomized controlled trials (RCTs) with a 3-year follow-up^17,27,28^ were included and jointly analysed meta-analysis presented functional recovery, active reincorporation, and anteroposterior and rotational stability.

Does the arthroscopic DB technique, compared to the arthroscopic SB technique, improve clinical outcomes in athletes?

Therefore, this meta-analysis aimed to determine the functional recovery and active reincorporation and the anteroposterior and rotational stability of patients undergoing anterior cruciate ligament (ACL) reconstruction using simple bundles (SB) or double bundles (DB).

## Results

### Study selection

**Figure 1:**
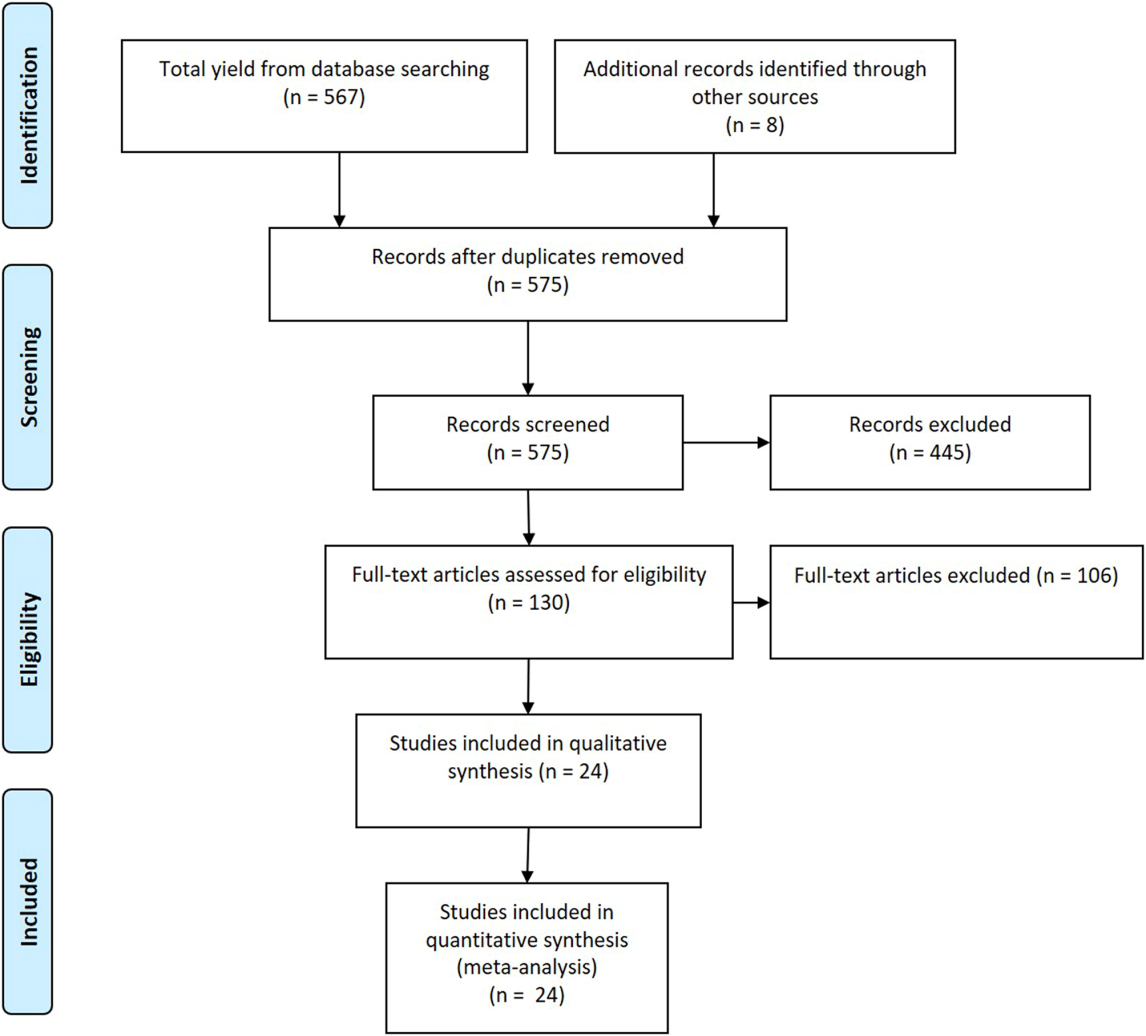
Study selection process. Our initial search of databases yielded 575 records. We searched the reference lists of relevant studies and related systematic reviews and found no additional records. After 445 records were excluded, 130 full-text articles were appraised. Thirty-eight were included in the qualitative synthesis and twenty-four were included in the quantitative analysis and meta-analyses. (CINAHL, Cumulative Index to Nursing and Allied Health Literature). Adapted from Moher et al.^65^.

The search yielded 575 records (Fig. 1), which were screened by 2 investigators, including 130 records which were assessed for eligibility.

### Qualitative systematic review

A total of 24 studies with 1707 patients were included in the qualitative systematic review^29–52^ (Table 1). All studies included patients undergoing ACL reconstruction. Of the studies, 22 used a randomized controlled trial design^29–33,35–44,46–52^, 1 used a nonrandomized design^34^ and 1 used a quasi-experimental design with 2 consecutive groups^45^. One group received single-bundle reconstruction while another group received double-bundle reconstruction of the ACL. In randomized controlled trials, both groups occurred at a 1:1 proportion. Six studies used semitendinosus autologous grafts for SB and DB reconstructions^29,33,45,46,48,52^ and 6 of them used both semitendinosus and gracilis grafts^32,35,43,44,49^. Three studies used tibial anterior allografts^38,39,47^, and one of them used frozen tibial anterior autologous allografts^47^. In 9 studies, the bone patellar tendon was used as the graft^30,31,34,40,41,46,49–51^. The outcomes presented in all studies were assessed in different follow-up periods with 13 short- and mid-term studies (less than 2 years)^30,31,33,34,42,43,45–50,52^, one preliminary study^48^, and 11 studies were long-term follow-ups (more than 2 years)^29,32,35–41,44,51^.

**Table 1:** Study characteristics.

Author, year	Level of Evidence	Sample	Single-bundle group	Double bundle group	Measures and outcomes
Adravanti et al.^1^	I	n = 60 Inclusion: age between 16 and 45 years, complete ACL rupture within 4 months	Single-bundle ACL reconstruction. The tibial tunnel was prepared using a dedicated elbow aimer in the posterior half of the native ACL footprint while maintaining the ACL stump. The femoral tunnel diameter was usually 7 to 8 mm and the tibial tunnel diameter 8 to 9 mm accordingly to the graft dimension. n = 30	Double-bundle ACL reconstruction. The semitendinosus was used for the anteromedial bundle and the gracilis for the posterolateral bundle. n = 30	Patients were evaluated preoperatively and after surgery at 6 months, 1, 3, and 6 years using the Lysholm score, IKDC form, and KT-2000
Ahlden et al.^3^	I	n = 103 Inclusion: patients with a unilateral ACL injury and older than 18 years	Single-bundle ACL reconstruction. The femoral tunnel was first addressed. The femoral ACL insertion site was marked with a Steadman awl in the shallow aspect of the AM bundle insertion site and near the centre of the ACL footprint. n = 50	Double-bundle ACL reconstruction. The femoral tunnels were first addressed. The femoral insertion sites of the AM and PL bundles were marked with a Steadman awl. n = 53	Clinical assessments at the preoperative and follow-up times were as follows: pivot-shift test, KT-1000 arthrometer laxity measurements, manual Lachman test, range of motion, Lysholm knee-scoring scale, and Tegner activity scale, KOOS, 1-legged hop test, and square hop test
Araki et al.^4^	I	n = 20 Inclusion: chronic ACL deficiency in one knee and had an indication for ACL reconstruction	Single femoral and single tibial tunnels were created at the central position between the original insertion of the AMB and PLB. n = 10	Two femoral and two tibial tunnels to reproduce the AMB and PLB. n = 10	KT-1000 measurements, isokinetic muscle peak torque, heel-height difference, and Lysholm score at the preoperative and one-year follow-up times between these two groups
Beyaz et al.^9^	I	n = 31 Inclusion: patients without lower limb bone fractures, who had not undergone previous lower extremity surgery, and whose other knee examination was normal	In the single-bundle method, the ACL was aligned in the middle of the tibial tunnel exit. n = 16	In the dual-bundle method, a 5 cm oblique incision was made 2 cm below and medial to the tibial tuberosity to harvest gracilis and semitendinosus tendons. n = 15	Clinical evaluations were performed at 8 years postoperatively with the IKDC, Tegner, and Lysholm knee-scoring systems
Bohn et al.^11^	I	n = 36 Inclusion: age 18–50 years, magnetic resonance imaging-verified ACL injury with symptoms of instability, no previous knee ligament surgery, no concomitant knee ligament injuries, and an uninjured contralateral knee	The tibial bone tunnel was positioned in the intercondylaris anterior area in the centre of the native tibial ACL footprint using the inner aspect of the lateral meniscus anterior insertion area as a landmark. n = 13	The semitendinosus tendon (for the AM bundle) and the gracilis tendon (for the PL bundle) were looped over a 20 mm EndoButton CL femoral fixation implant. n = 23	The tibial rotation was determined during walking, running, and a pivoting task. Other outcome parameters were KT-1000 knee laxity measurements and subjective outcome scores of KOOS and IKDC
Ebert et al.^20^	II	n = 50 Inclusion: less than 60 years old, non-cartilage lesions above grade 3 or lower than 3 cm^2^, knee joint dislocations and partial resection of the meniscus of less than 50%	For the SB technique, a graft that was 7–9 mm in diameter and 7–9 cm in length was prepared by folding it. n = 31	Two grafts were prepared for the DB technique. n = 19	The VAS for pain and function, the Tegner activity score, IKDC and the Lysholm and Marshall scores were used as evaluation methods; the anterior stability (KT-1000 arthrometer measurement) and the deficits in muscle strength in extension and flexion of both knees were measured in a standardized manner one year after surgery
Fujita et al.^22^	I	n = 55 Inclusion: the patient was diagnosed with ACL insufficiency and provided informed written consent for this study	Double semitendinosus combined with double gracilis tendons for the AM bundle. n = 31	The PL bundle graft in DB reconstruction and combined quadruple semitendinosus and double gracilis tendons in the AM and PL reconstructions; 2 femoral and 2 tibial tunnels to reproduce the AM and PL bundles for DB reconstruction were created. n = 19	The Lysholm score, Tegner score, anterior laxity with the KT-1000 arthrometer, rotator instability with the pivot-shift test, and muscle strength with knee extensor and flexor isokinetic peak torques at 60°/s were evaluated
Hussein et al.^26^	I	n = 209 Inclusion: an ACL rupture in active patients with a closed growth plate	The procedure of anatomic single-bundle reconstruction was similar to anatomic double-bundle reconstruction. n = 78	With the scope in the medial portal, a 3/32 Steinman pin was introduced through the accessory medial portal and placed at the centre of the PL femoral insertion site. In chronic cases, we placed it below the lateral intercondylar ridge and anterior to the bifurcate ridge. n = 138	The outcomes were the Lysholm score and subjective IKDC form. The KT-1000 arthrometer was used to evaluate anteroposterior stability, and the pivot-shift test was used to determine rotational stability
Jarvela et al.^28^	II	n = 90 Inclusion: primary ACL reconstruction, closed growth plates, and the absence of ligament injuries to the contralateral knee	The femoral tunnel was drilled through an anteromedial portal as posterior as possible without breaking the posterior wall of the femur with a free-hand technique at approximately 10 o’clock in the right knee and 2 o’clock in the left knee. n = 60	Two tunnels on the femoral side were made via an anteromedial portal (not transtibial) with a free-hand technique without a guide to the anatomic position of the insertion sites of each bundle. n = 30	The evaluation methods consisted of a clinical examination, which included stability measurements using a KT-1000 arthrometer, and a manual pivot-shift test. The IKDC and Lysholm knee scores were used to evaluate the knee preoperatively and at the 10-year follow-up
Kang et al.^30^	II	n = 84 Inclusion: (1) no history of previous surgery in the injured knee; (2) no concomitant injury of other knee ligaments; (3) a healthy contralateral knee; (4) chondral lesions no more severe than grade II according to the Outerbridge classification; (5) meniscus repair or partial meniscectomy involving less than one-third of the entire meniscus; (6) no patellofemoral symptoms or absence of systemic illnesses	Single-bundle reconstruction with modified BPTB allograft was shaped into a column of 25 mm in length and 10 mm in diameter; n = 43	For DB ACL reconstruction, tibialis anterior allografts were prepared to make 2 double-looped grafts for the AM and PL bundles. n = 41	Clinical outcomes including Lachman and pivot-shift tests, KT-1000 arthrometer measurements, and IKDC classification; Lysholm and Tegner activity scores were compared between the two groups at the last follow-up
Karikis et al.^31^	I	n = 105 Inclusion: patients > 18 years old with a unilateral ACL injury	The femoral tunnel was addressed first. The femoral ACL insertion site was marked with an awl in the shallow aspect of the AM bundle insertion site, which is near the centre of the ACL footprint, to place the centre of the tunnel just as deep as the bifurcate ridge approximately 8 to 10 mm from the posterior cartilage at the 3 or 9 o’clock position in the notch orientation and with the knee at 90° of flexion. n = 52	For the DB technique, both femoral and tibial remnants of AM and PL bundles were identified with the knee at 90° of flexion. The femoral tunnels were addressed first. The femoral insertion sites of the AM and PL bundles were identified and marked with an awl. n = 53	Multiple subjective and objective clinical evaluation tests and radiographic assessments of osteoarthritis (OA) were performed including the following: the Tegner score, the pivot-shift test, KT-1000 arthrometer laxity measurements, manual Lachman test, single-legged-hop test, square-hop test, range of motion, Lysholm knee scoring scale, Tegner activity scale, or Knee injury and Osteoarthritis Outcome Score
Koga et al.^32^	II	n = 53 Inclusion: primary ACL reconstruction with an autologous semitendinosus tendon	For the SB reconstruction, 2 double-strand grafts were looped and hooked to 1 EndoButton CL. n = 25	For DB reconstruction, 2 double-strand bundles for the anteromedial bundle (AMB) and posterolateral bundle (PLB) were created with the EndoButton CL devices. The open end of each graft was closed in the same fashion as the SB method. n = 28	The following evaluation methods were used: clinical examination, KT-1000 arthrometer measurement, muscle strength, Tegner activity score, Lysholm score, subjective rating scale regarding patient satisfaction and sports performance level, graft retear, contralateral ACL tear, and additional meniscus surgery
Liu et al.^34^	I	n = 80 Inclusion: complete, isolated, chronic ACL lesions (mean injury-to-surgery interval, 23.5 months; range, 1.5–180 months) received an ACL reconstruction with a 6- to 8-stranded HG	The femoral tunnel was drilled through the AAMP behind the resident’s ridge as posterior as possible without breaking the posterior wall of the femur and using a 6-mm femoral guide at approximately the 10 o’clock (or 2 o’clock) position. n = 40	On the femoral side, both the AMB and PLB tunnels were drilled through the AAMP behind the resident’s ridge as posterior as possible without breaking the posterior wall of the femur and using a 6-mm femoral guide. n = 40	The outcome assessment was performed by a blinded independent observer using International Knee Documentation Committee (IKDC), Tegner, and Lysholm scores as well as range of motion (ROM), Lachman test, pivot-shift test, KT-2000 arthrometer side-to-side difference, and return-to-sport data
Liu et al.^35^	I	n = 42 Inclusion: 1. Men aged 18–40 at the time of surgery; 2. First ACL reconstruction surgery; 3. Single leg involvement; and 4. Able to attend preoperative assessment	For HT-SB surgery, the semitendinosus and gracilis tendons (approximately 7–9 mm in diameter) were harvested and inserted into the femoral and tibial tunnels (both approximately 7–9 mm in diameter). n = 22	For HT-DB surgery, the semitendinosus and gracilis tendons were harvested. Two tunnels (6–7 mm in diameter for the AM tunnel and 5–7 mm in diameter for the posterolateral (PL tunnel)) were drilled over both the femur and tibia. n = 20	The KT-1000, Lysholm, IKDC, one-leg hop test and Lachman test were performed blindly at baseline and 1-year post-reconstruction
Mayr et al.^37^	I	n = 64 Inclusion: all consecutive patients presented to the outpatient clinic with an ACL rupture	For SB ACL reconstruction, both tendons were used as a 4-strand graft; for DB reconstruction, the gracilis tendon was used as a double-strand graft to replace the anteromedial bundle and the double-strand semitendinosus tendon was used for replacement of the posterolateral bundle. n = 30	In the DB technique, the femoral drill pin for the anteromedial bundle was placed into the proximal and anterior part of the femoral footprint of the ACL and for the posterolateral bundle was placed into the posterior and distal portion. n = 34	A follow-up examination 2 years after surgery consisted of IKDC 2000 assessment, Laxitester measurement of anteroposterior translation regarding rotational stability, and radiographic evaluation
Mayr et al.^38^	I	n = 64 Inclusion: all consecutive patients presented to the outpatient clinic with an ACL rupture	For SB ACL reconstruction, both tendons were used as a 4-strand graft; for DB reconstruction, the gracilis tendon was used as a double-strand graft to replace the anteromedial bundle and the double-strand semitendinosus tendon was used for replacement of the posterolateral bundle. n = 30	In the DB technique, the femoral drill pin for the anteromedial bundle was placed into the proximal and anterior part of the femoral footprint of the ACL and for the posterolateral bundle was placed into the posterior and distal portion. n = 34	A follow-up examination 5 years after surgery consisted of IKDC 2000 assessment, Laxitester measurement of anteroposterior translation regarding rotational stability, and radiographic evaluation
Misonoo et al.^42^	II	n = 44 Inclusion: patients whose ACL was reconstructed using either a SB o rDB method	For the SB reconstruction, the semitendinosus tendon was used as two double stranded grafts. First, using a tibial guide, the tibial tunnel was created at the centre of the ACL footprint. n = 22	In the technique used for DB reconstruction, two femoral and two tibial tunnels were created under controlled arthroscopic visualization to anatomically reproduce both the AM and PL bundle using the hamstring tendon graft. n = 22	Clinical assessment, including Tegner score, Lysholm score, and knee arthrometric measurement, revealed a restoration of the reconstructed knee stability
Sasaki et al.^49^	I	n = 14 Inclusion: unilateral ACL reconstruction	Single-bundle ACL reconstruction with Patellar Tendon: either the modified transtibial technique or the transportal technique was selected during surgery depending on accessibility to the femoral ACL insertion. A 10 mm-wide bone-patellar tendon-bone graft was harvested from the central portion of the patellar tendon with approximately 15 mm–long bone plugs on both ends. n = 5	Double-bundle ACL Reconstruction with Hamstring Tendon: the semitendinosus tendon was usually harvested with a tendon harvester. The distal and proximal half of the semitendinosus tendon was looped and used as the AMB and PLB graft, respectively. n = 9	Clinical outcomes (knee flexion (ROM), heel-height difference, side-to-side difference in anterior laxity, rotational laxity, and Tegner activity score) were compared between the DB and SB groups and an examination of factors affecting subjective outcomes (KOOS results) was performed
Song et al.^50^	II	n = 130 Inclusion: patients with ACL injury, chondral lesions less than the Outerbridge grade of 3, and with or without meniscal injury	For the SB ACLR, the tibialis anterior allograft was also prepared as a single-looped graft (diameter, 8–9 mm). After tibial tunnel preparation at the centre of the ACL insertion, a femoral tunnel at the centre of the footprint was created through the anteromedial portal. n = 65	For DB reconstruction, fresh-frozen tibialis anterior allografts were prepared to make 2 single-looped grafts of 6-mm diameter for PLB and of 7-mm diameter for AMB. n = 65	The stability results were evaluated using the Lachman and pivot-shift tests and stress radiography. Additionally, the functional outcomes were based on the Lysholm knee score, Tegner activity score, and IKDC subjective scale
Ventura et al.^54^	II	n = 80 Inclusion: 18 to 45 years old; primary ACL reconstruction; absence of concomitant cartilage, ligament, or meniscal pathology requiring surgery; and no history of knee injury or lower limb pathology	Patients belonging to the SB group underwent SB reconstruction with doubled hamstrings. n = 40	Patients belonging to the DB group underwent DB reconstruction using a 2-stranded semitendinosus tendon for the AM bundle and a 2-stranded gracilis tendon for the PL bundle. n = 40	Patients were assessed preoperatively with functional assessment including the International Knee Documentation Committee 2000 knee subjective form and visual analogue scale as well as physical examination (including the pivot-shift test and instrumented knee laxity measurement). Vertical jump assessment with the Optojump system has been introduced as a method comparing functional ability between the 2 surgical techniques. The same protocol was repeated at 6 months, 12 months, and 2 years after surgery
Volpi et al.^55^	II	n = 40 Inclusion: specific sports activities age 18–45, no additional ligamentous lesions, absence of rheumatic pathologies, type IV Outerbridge chondral lesions, axial deviation of the knee, and any previous surgery to the examined knee	Single-bundle ACL reconstructions with the patellar tendon were performed using two re-absorbable cross pins for the femoral fixation and both tibial rigid fix and re-absorbable pins for the tibial fixation. n = 20	Double-bundle ACL reconstruction with semitendinosus and gracilis tendons were performed using the transtibial technique with a dedicated guide. The femoral fixation of both PL and AM bundles was achieved with pins, while for the tibial side, both bundles were fixed with a metal staple or bioscrew at 108° and 45–50° of flexion, respectively. n = 20	Clinical assessment, including Tegner score, Lysholm score, IKDC and KT-1000
Xu et al.^58^	I	n = 80 Inclusion: primary ACL rupture in adult patients	The procedure was similar to the anatomic double-bundle reconstruction. The femoral tunnel was also created through the accessory medial portal, but the centre of the tunnel was placed in the middle of the insertion site. n = 40	The AM and PL tunnels on the femur were drilled based on the identified insertion sites through the accessory medial portal. n = 40	Pre- and post-operatively, all patients received a preoperative examination, including Lachman, anterior drawer, and pivot shift testing, and were also tested with KT-1000 arthrometer with a knee flexion of 30 and 90° and a manual maximum force. All patients were also evaluated with the IKDC subjective score, Lysholm score and Tegner score
Zaffagnini et al.^60^	I	n = 79 Inclusion: positive clinical examination with (Lachman test, anterior drawer test and pivot-shift test) respect to a contra-lateral normal knee. Patients with medial and lateral meniscal injuries, grade 1 or 2 MCL injuries and Outerbridge 1 or 2 chondral lesions were also included	Autologous LSBPTB technique: BPTB autograft was harvested through a single straight midline incision. In all cases, we used the central third of the ipsilateral patellar tendon. n = 39	naDBH technique: Semitendinosus and gracilis tendons from the ipsilateral limb were harvested with an open tendon stripper. n = 40	Patients were subjectively and objectively evaluated using the IKDC score, Tegner level, and manual maximum displacement test with a KT-2000TM arthrometer. Radiographic evaluation was performed according to the IKDC grading system and the re-intervention rate for meniscal lesions was also recorded
Zhang et al.^61^	I	n = 94 Inclusion: primary ACL reconstruction with no combined PCL injury, lateral collateral ligament injury, PL rotatory instability or fracture about knee joint, no subtotal or total meniscectomy, no previous knee ligament surgery, no arthritic changes, no malalignment and a normal contralateral knee	In single-bundle reconstruction, a tibial tunnel was first made by inserting a 2.0 Kirschner wire into the centre of ACL insertion to the tibia and then drilling with a cannulated drill and a dilatar to create a bone tunnel with the same diameter as the tendon graft. n = 49	In double-bundle reconstruction, a 2.0 Kirschner wire was inserted posterior to the footprint of ACL insertion into the tibia via the Pro-trae ACL guide system; then, a dilatar and a cannulated drill were used to create a bone tunnel with the same diameter as the PL bundle of the graft. n = 45	The rotational stability, as evaluated by the pivot-shift test, was significantly superior in Group DB compared to that in Group SB. No significant difference regarding ACL revisions, total flexion work, mean peak flexion torque and extension work between the groups was detected using the Tegner activity score, the knee injury and osteoarthritis outcome score, the Lysholm functional score, anterior knee pain or mobility, and subjective knee function. In addition, the Lachman test or the KT-1000 maximum manual force test was investigated

*ACL* anterior cruciate ligament, *ACLR* anterior cruciate ligament reconstruction, *AM* anteromedial, *AMB* anteromedial-bundle, *BPTB* bone-patellar tendon-bone, *DB* double-bundle, *HG* human givens, *IKDC* international Knee Documentation Committee, *KOOS* Knee injury, osteoarthritis and outcome score, *LBPTB* lateralized single-bundle bone-patellar tendon-bone, *MCL* medial collateral ligament, mm millimetres, *NaDBH* Non-anatomical autologous double-bundle, *PCL* posterior cruciate ligament, *PL* posterolateral, *PLB* posterolateral-bundle, *ROM* range of motion, *SB* single-bundle, *VAS* visual analogue scale.

In the presented outcomes, most of the studies showed no differences; however, in 2 studies, the pivot-shift test showed better results in the double-bundle group as shown in (*P* < 0.001)^40^ and (*P* = 0.003)^51^. Two studies^47,49^ showed better grades of objective and subjective IKDC scores and presented high heterogeneity between the IKDC score objective studies. Koga et al.^40^ showed better results in the double-bundle group (*P* = 0.024) in the Lachman test, and KT measurements were better in the double-bundle group (mean, 1.4 mm vs. 2.7 mm; *P* = 0.0023). The Tegner score was also better in the double-bundle group (*P* = 0.033). Zaffagnini et al.^51^ showed that the double-bundle hamstring group had a significantly higher Tegner level (*P* = 0.0007) and a higher passive range of motion recovery (*P* = 0.0014). The side-to-side difference in posterior translation decreased in the double-bundle group with a significant difference between the 2 groups (*P* < 0.05).

### Assessment of risk of bias

**Figure 2:**
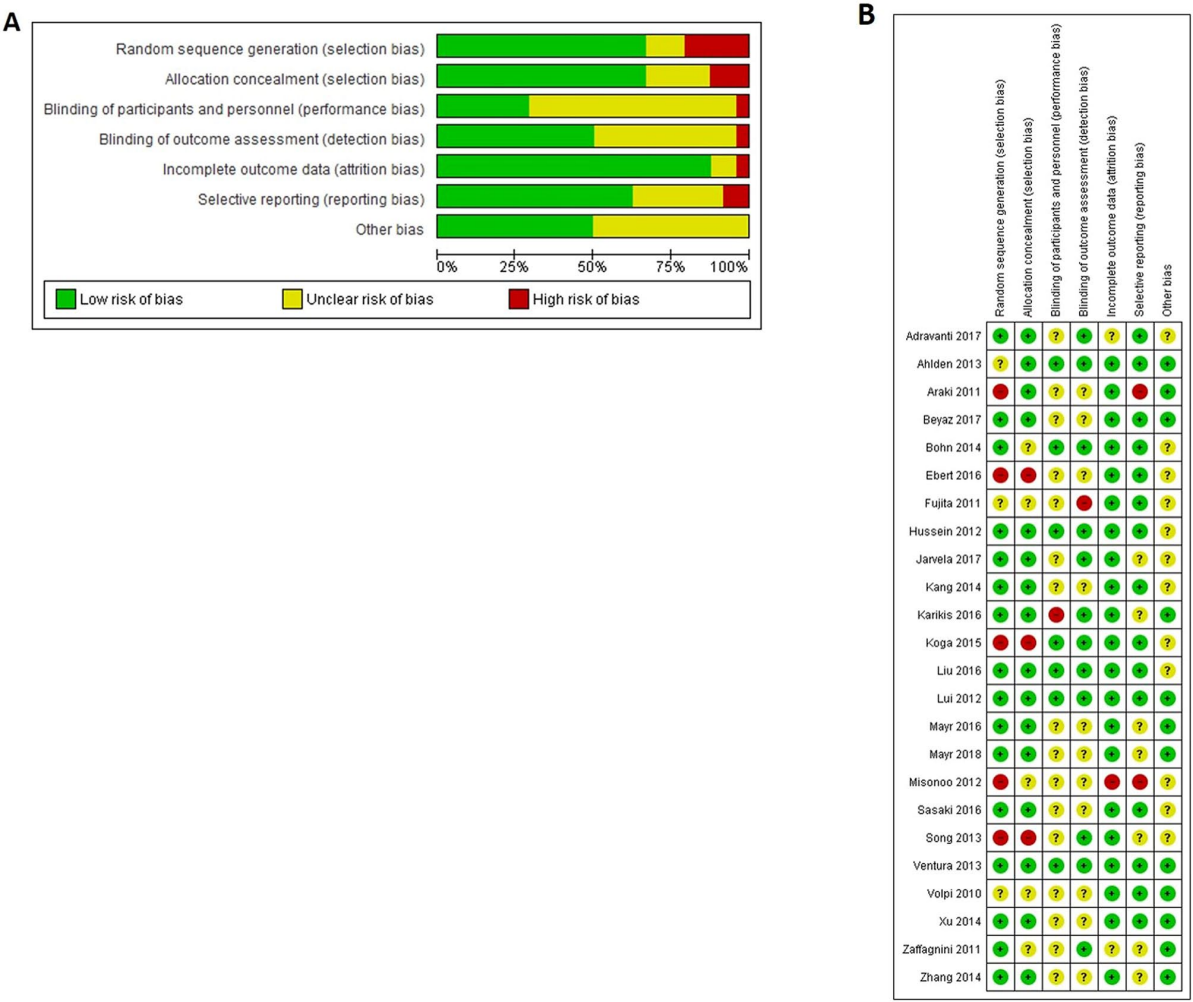
(**A**) Risk of bias within the included studies (24 studies, 1707 patients). (**B**) Risk of bias across included studies. The risk of bias was unclear for most of the studies. Data collected from RevMan 5.3 software.

The risk of selection bias, related to a lack of random sequence generation and allocation concealment, was high in 5 studies using non-random group allocation^31,34,40,45,47^. Four studies used random allocation, but the method was unclear^30,35,49,51^, whereas the remainder used a random-numbers table^29,32,33,36,38,39,41,43,44,46,48,50,52^. Seven studies reported blinding of participants, personnel, and outcome assessors^30,33,36,42,48^, whereas in 2 studies, only the outcome assessors were blinded^39,51^. Blinding procedures were not reported in half of the studies^31,32,34,38,43–46,49,50,52^. The risk of attrition bias was deemed high in only 1 study because they lost participants in the follow-up that were needed for analysis^45^. The risk of reporting bias was unclear in half of the meta-analyses^29,33–38,40,41,45–47^ (Fig. 2).

### Heterogeneity

We evaluated the clinical heterogeneity of 24 studies. Statistical heterogeneity was calculated for both continuous (Lysholm and Tegner score, internal rotation range, KT-1000/2000, and subjective IKDC score) and dichotomous (pivot shift, Lachman test, and objective IKDC score) variables.

In the 3 studies included in the internal rotation range, the heterogeneity was high (I^2^ = 73%)^43,44,48^. Heterogeneity was also high for the 6 studies included in the objective IKDC score (I^2^ = 91%)^29,36,37,43,44,49^ and for the 12 studies included in the pivot shift test (I^2^ = 64%)^30,31,36–40,44–47,50^. Among the 12 studies included in KT-1000/2000, the heterogeneity was moderate (I^2^ = 40%)^29–31,33–37,39,40,42,45^. Heterogeneity was low for 13 studies included in the Lysholm score (I^2^ = 42%)^29,31–37,39–41,50,52^ and for 13 studies included in the Tegner score (I^2^ = 8%)^32–35,39–41,45–47,49,50,52^. Heterogeneity was also low for 9 studies included in the subjective IKDC score (I^2^ = 0%)^32,33,36,41,43,44,48,50^ and for 8 studies included in the Lachman test (I^2^ = 1%)^30,31,33,38,39,43,47,52^. No studies were excluded due to a high risk of bias that could influence the presented heterogeneity.

### Quantitative meta-analyses

**Figure 3:**
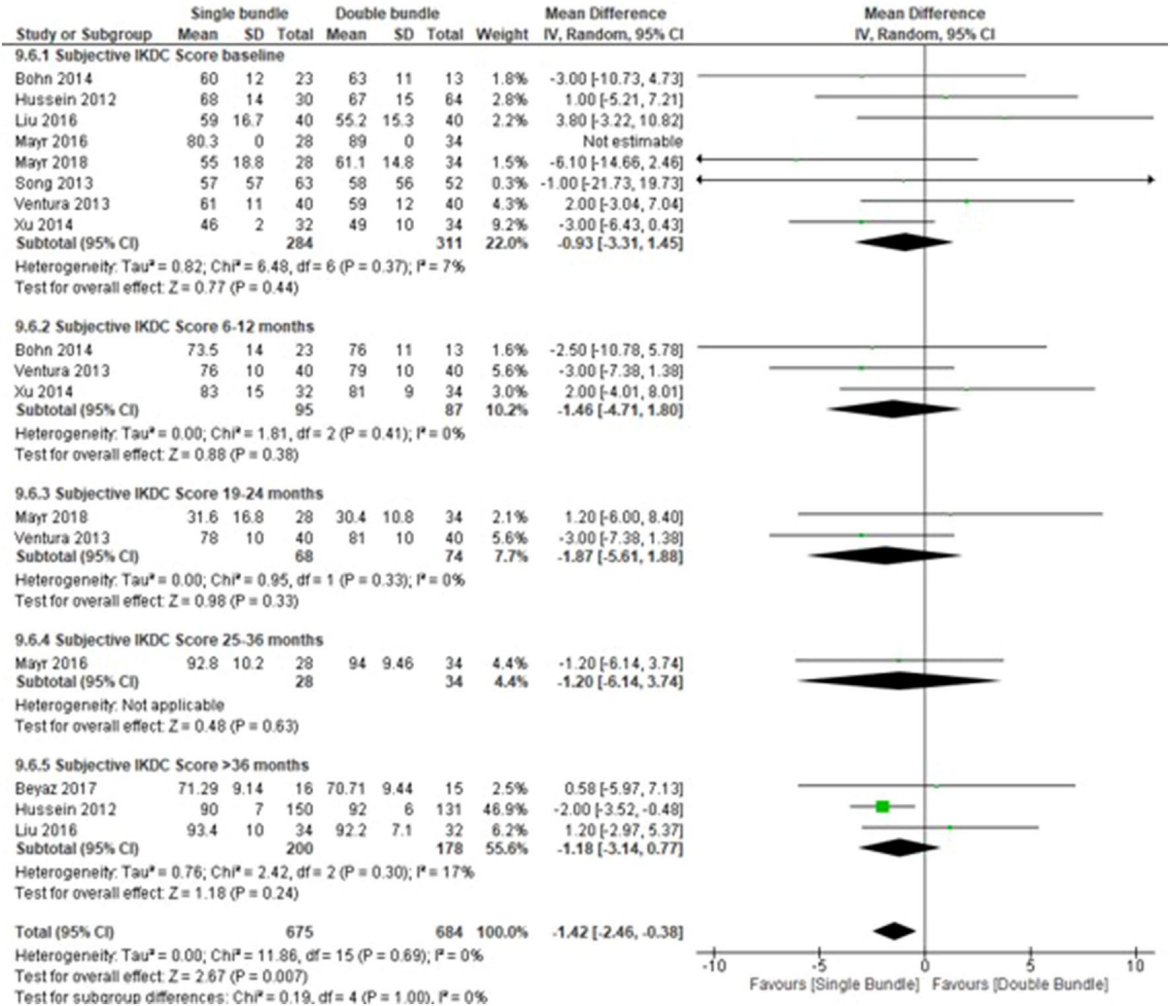
Forest plots of knee functionality data outcomes for single-bundle versus double-bundle reconstruction. Weights are from random-effects analysis. (**A**) Continuous data for the Subjective IKDC score (8 studies in the baseline period, 654 patients; 4 studies in the 6–12-month period, 150 patients; 1 study in the 19–24-month period, 78 patients; 2 studies in the 25–36-month period, 101 patients and 5 studies in the > 36-month period, 536 patients). Heterogeneity was low: τ^2^ = 0.00; χ^2^ = 11.86, df = 15 (*P* = 0.69); I^2^ = 0% (CI, confidence interval; IV, inverse variance). (**B**) Dichotomous data for the objective IKDC score (3 studies in the baseline period, 280 patients; 1 study in the 19–24-month period, 62 patients; 2 studies in the 25–36-month period, 102 patients and 1 study in the > 36-month period, 70 patients). The heterogeneity was high: τ^2^ = 0.02; χ^2^ = 69.84, df = 6 (*P* < 0.001); I^2^ = 91% (CI, confidence interval; M-H, Mantel–Haenszel). Data collected from RevMan 5.3 software.

**Figure 4:**
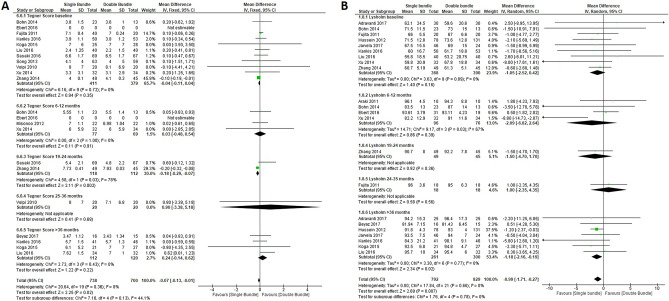
Return to pre-injury level data forest plot outcomes for single-bundle versus double-bundle reconstruction. Weights from fixed effects for the Tegner score analysis and random effects for the Lysholm score analysis are presented. (**A**) Continuous data for the Tegner score (10 studies in the baseline period, 790 patients; 4 studies in the 6–12-month period, 146 patients; 2 studies in the 19–24-month period, 230 patients; 1 study in the 25–36-month period, 40 patients and 4 studies in the > 36-month period, 232 patients). Heterogeneity was low: χ^2^ = 20.64, df = 19 (*P* = 0.36); I^2^ = 8% (CI, confidence interval; IV, inverse variance). (**B**) Continuous data for the Lysholm score (9 studies in the baseline period, 758 patients; 4 studies in the 6–12-month period, 172 patients; 1 study in the 19–24-month period, 94 patients; 1 study in the 25–36-month period, 36 patients and 7 studies in the > 36-month period, 561 patients). Heterogeneity was low: τ^2^ = 0.00; χ^2^ = 17.84, df = 6 (*P* = 0.66); I^2^ = 0% (CI, confidence interval; IV, inverse variance). Data collected from RevMan 5.3 software.

**Figure 5:**
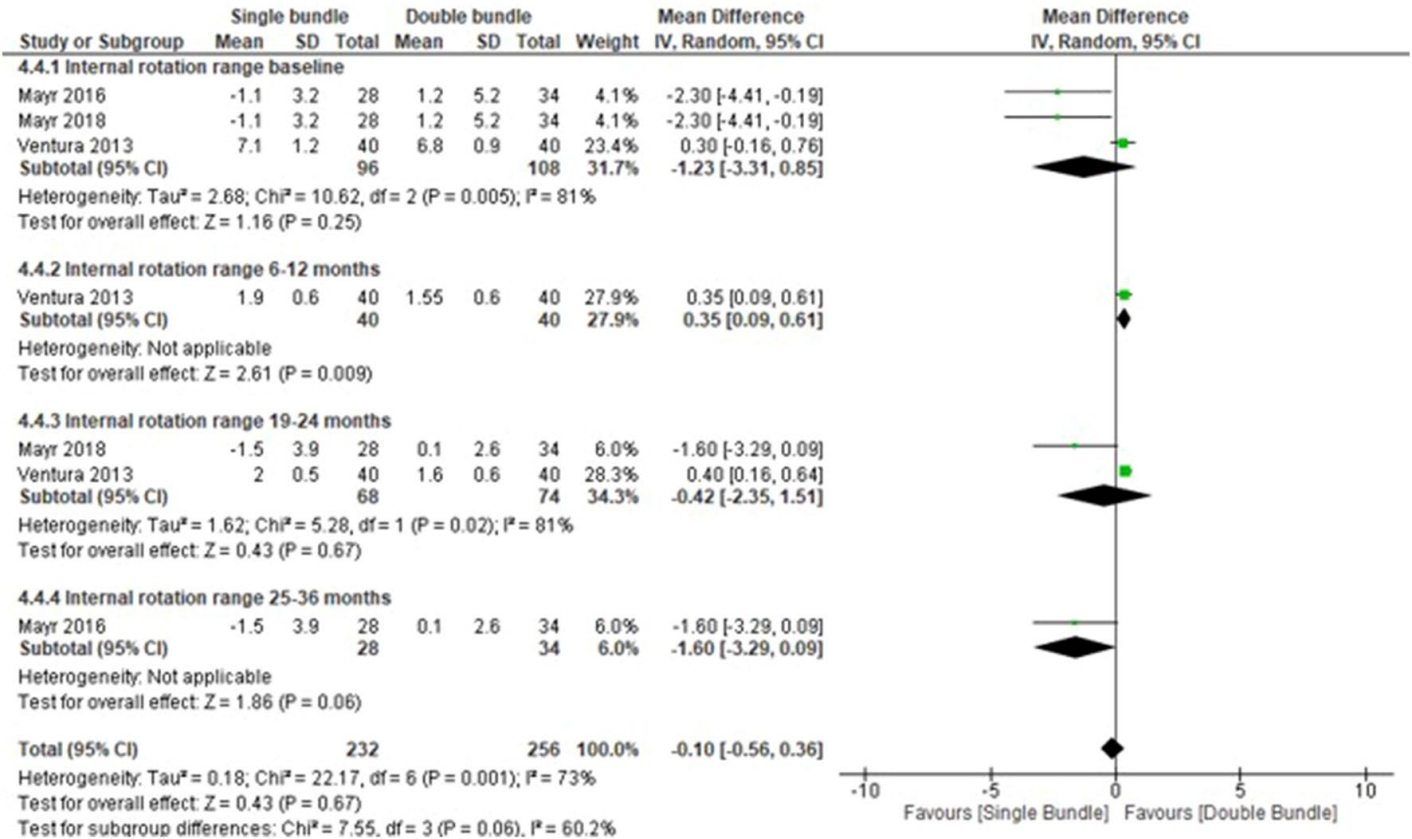
Rotational knee stability data forest plot outcomes for single-bundle versus double-bundle reconstruction. Weights are from random-effects analysis. (**A**) Dichotomous data for the pivot shift test (9 studies in the baseline period, 842 patients; 4 studies in the 6–12-month period, 242 patients; 3 studies in the 19–24-month period, 296 patients; 1 study in the 25–36-month period, 84 patients and 5 studies in the > 36-month period, 493 patients). The heterogeneity was high: τ^2^ = 0.00; χ^2^ = 58.24, df = 21 (*P* =  < 0.001); I^2^ = 64% (CI, confidence interval; M-H, Mantel–Haenszel). (**B**) Continuous data for the internal rotation range (3 studies in the baseline period, 204 patients; 1 study in the 6–12-month period, 80 patients; 2 studies in the 19–24-month period, 142 patients; and 1 study in the 25–36-month period, 62 patients). Heterogeneity was high: τ^2^ = 0.18; χ^2^ = 22.17, df = 6 (*P* < 0.001); I^2^ ¼ 73% (CI, confidence interval; IV, inverse variance). Data collected from RevMan 5.3 software.

**Figure 6:**
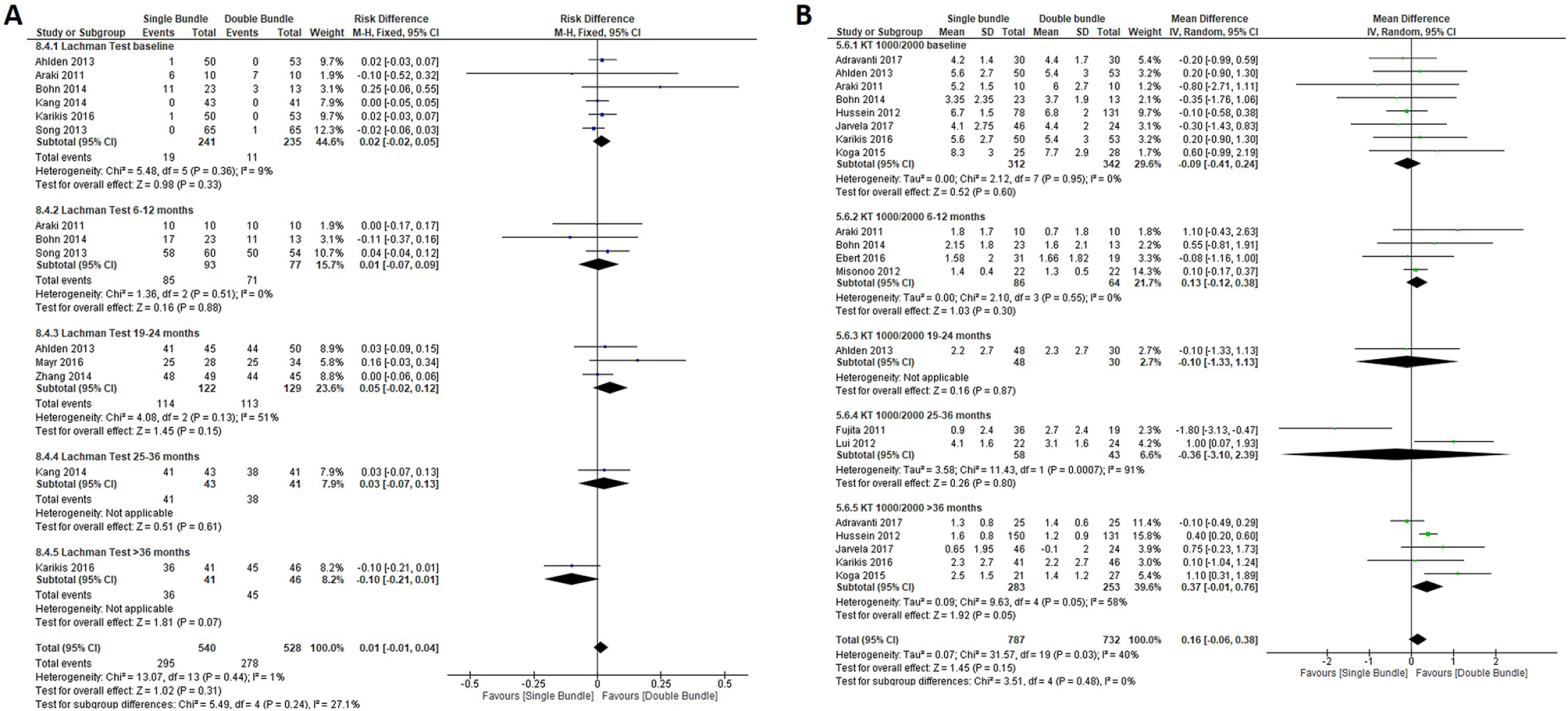
Forest plots of knee anterior stability data for single-bundle versus double-bundle reconstruction. Weights are from fixed effects for the Lachman test analysis and random effects for the KT 1000/2000 analysis. (**A**) Dichotomous data for the Lachman test (6 studies in the baseline period, 476 patients; 3 studies in the 6–12-month period, 170 patients; 3 studies in the 19–24-month period, 251 patients; 1 study in the 25–36-month period, 84 patients and 1 study in the > 36-month period, 87 patients). The heterogeneity was low: χ^2^ = 13.07, df = 13 (*P* = 0.44); I^2^ = 1% (CI, confidence interval; M-H, Mantel–Haenszel). (**B**) Continuous data for KT-1000/2000 (8 studies in the baseline period, 654 patients; 4 studies in the 6–12-month period, 150 patients; 1 study in the 19–24-month period, 78 patients; 2 studies in the 25–36-month period, 101 patients and 5 studies in the > 36-month period, 536 patients). Heterogeneity was moderate: τ^2^ = 0.07; χ^2^ = 31.57, df = 19 (*P* = 0.03); I^2^ = 40% (CI, confidence interval; IV, inverse variance). Data collected from RevMan 5.3 software.

A total of 24 studies with 1707 patients were included in the quantitative meta-analyses. We grouped studies for statistical analyses based on follow-up into the following categories: baseline, 6–12 months, 13–18 months, 19–24 months, 25–36 months and > 36 months. The aforementioned categories were used in each of the following outcome measures: Lysholm score (13 studies [5 subgroups] [22 comparisons], baseline period, n = 758 patients; 6–12 month period, n = 172 patients; 19–24 month period, n = 94 patients; 25–36 month period, n = 36 patients and > 36 month period, n = 561 patients); Internal rotation range (3 studies [4 subgroups] [7 comparisons], baseline period, n = 204 patients; 6–12 month period, n = 80 patients; 19–24 month period n = 142 patients and 25–36 month period, n = 62 patients); KT/1000–2000 (12 studies [5 subgroups] [20 comparisons], baseline period, n = 654 patients; 6–12 month period, n = 150 patients; 19–24 month period, n = 78 patients; 25–36 month period, n = 101 patients and > 36 month period, n = 536 patients); Tegner score (13 studies [5 subgroups] [22 comparisons], baseline period, n = 790 patients; 6–12 month period, n = 146 patients; 19–24 month period, n = 230 patients; 25–36 month period, n = 40 patients and > 36 month period, n = 232 patients); Subjective IKDC score (9 studies [5 subgroups] [17 comparisons], baseline period, n = 654 patients; 6–12 month period, n = 150 patients; 19–24 month period, n = 78 patients; 25–36 month period, n = 101 patients and > 36 month period, n = 536 patients); Lachman test (8 studies [5 subgroups] [14 comparisons] (baseline period, n = 476 patients; 6–12 month period, n = 170 patients; 19–24 month period, n = 251 patients; 25–36 month period, n = 84 patients and > 36 month period, n = 87 patients). Regarding continuous variables, the subjective IKDC score was lower in the SB group than in the DB group (mean difference, − 1.42; 95% CI − 2.46 to − 0.38; *P* = 0.007) (Fig. 3)^32,33,36,41,43,44,48,50^ and the Tegner score (mean difference, − 0.07; 95% CI − 0.13 to − 0.01; *P* = 0.02), which favoured SB in both outcomes (Fig. 4 A)^32–35,39–41,45–47,49,50,52^. In the internal rotation range, no differences were found between the two groups (mean difference, − 0.10 mm; 95% CI − 0.56 mm to 0.36 mm; *P* = 0.67)^43,44,48^ (Fig. 5) as KT/1000–2000 (mean difference, 0.16; 95% CI − 0.06 to 0.38; *P* = 0.15)^29–31,33–37,39,40,42,45^ (Fig. 6 B) (Table 2).

**Table 2:** Summary of meta-analyses of continuous variables.

Outcome	No. of Studies	Mean Differences (95% CI)	*P*
Lysholm score for single-bundle vs. double-bundle	13	− 0.99 (− 1.71 to − 0.27)	0.007
Subjective IKDC score for single-bundle vs. double-bundle	9	− 1.42 (− 2.46 to − 0.38)	0.007
Tegner score for single-bundle vs. double-bundle	13	− 0.07 (− 0.13 to − 0.01)	0.02
Internal rotation range for single-bundle vs. double-bundle	3	− 0.10 (− 0.56 to 0.36)	0.67
KT-1000/2000 for single-bundle vs. double-bundle	12	0.16 (− 0.06 to 0.38)	0.15

*CI* confidence interval, *IKDC* International Knee Documentation Committee Knee Form.

Regarding dichotomous variables, the Lachman test percentage no was higher in the double-bundle group than in the single-bundle group (RD, 0.01; 95% CI − 0.01 to 0.04; *P* = 0.13)^30,31,33,38,39,43,47,52^ (Fig. 6 A) (Table 3).

**Table 3:** Summary of meta-analyses of dichotomous variables.

Outcome	No. of studies	Risk Difference (95% CI)	*P*
Lachman test for single-bundle vs. double-bundle	8	0.01 (− 0.01 to 0.04)	0.13

*CI* confidence interval.

Data on the re-rupture rate in both techniques were not evaluated in the included studies.

## Discussion

In this meta-analysis, we attempted to show whether there were significant differences between SB and DB interventions in the recovery of functionality after ACL tears, since previous studies did not demonstrate this result strongly enough. We found significant differences favourable to DB reconstruction in the return to the preinjury level according to the Lysholm score (*P* = 0.007) and the functionality of the knee according to the IKDC subjective score (*P* = 0.007).

Residual instability in reconstructive ACL surgery is the main cause of mechanical failure. Techniques that reduce this instability, mainly in the rotational plane, have been previously described and highlight those that involve the performance of a DB to reproduce the original anatomy of the ACL in the anteromedial (AM) and posterolateral (PL) bundles by arthroscopic surgery.

There are some differences between the technical aspects and tips but the real difference between the whole DB reconstruction surgery is the realization of an only tibial tunnel or double tibial tunnel as well, since all the DB are with two tunnels. Respect to the SB surgery it would come based on the use of the new concept of anatomical technique (or through anteromedial portal) or classic through transtibial, being a relatively new technique^53,54^. Mostly of the studies included in our systematic review showed highlight an improvement in rotational stability (based on exploratory manoeuvres) throughout the follow-up according to functional reincorporation by using DB reconstruction surgery.

This study was conducted to compare the early-, mid-, and long-term follow-up of patient operations using DB and SB techniques. The results show that there is no clear or significant difference in the clinical stability and knee function or in sports incorporation with the true difference being the subjective assessment by the patient. These results are consistent with those presented by another recent systematic review and adds subjective assessment data to previous datasets^55^. The non-differences in the previous laxity do not confirm previous findings. Björnsson et al.^56^ shows results with DB improvement, but this systematic review presents RCT, prospective and retrospectives studies with less evidence regarding this meta-analysis and in the context of rotational stability. Our findings stand out with a non-significant improvement (internal rotation and pivot shift), which seems to be in contrast with the technical gesture of adding a PL fascicle to the conventional technique. Perhaps the classic concept of injury of "the other" peripheral structures, such as the collateral ligaments and the muscles that cross the joint and play an important role in the concept of rotational stability^55,57^.

In Kongtharvonskul et al. study, clinical function showed a significant statistical difference between the DB and SB techniques in autologous ACL reconstruction^58^, results similar to those found in our meta-analysis.

The strengths of results present in this works are: 1. Although there do not seem to be any functional differences from the immediate postoperative period, in the return to physical and sports activity differences are shown at 18 months after the DB, which are also evident after 3 years for the Lysholm and Tegner score (Fig. 4). For sports, this is of the utmost importance and often not shown during these mid-term and long-term follow-ups, according to results shown by Xu et al.^10^. 2. Subjective assessment through the IKDC shows a higher score with a better feeling of stability from the subjective viewpoint in the mean score of the total in the DB vs. SB technique, which is the most statistically significant for the outcomes of the study. This analysis of the subjective feeling of the patient in favour of the DB technique allows the patient greater confidence in their return to physical activities in patients undergoing the DB technique. Furthermore, this disagrees with previous studies that found no differences in subjective outcome measures between DB and SB reconstruction^10^. These results were not seen in the objective IKDC score, but there was a significant and favourable trend toward the DB technique observed (*P* = 0.10) (Fig. 3), which agrees with Xu et al.’s meta-analysis^10^ and concludes that DB anterior cruciate ligament reconstruction resulted in significantly better anterior and rotational stability and higher IKDC objective scores than SB reconstruction. Therefore, the evaluation of the patient and activity levels could be the strength of choice when choosing the DB technique. 3. Lachman anteroposterior instability shows similar results in both techniques, which indicates that there is no advantage for any technique from the medical anteroposterior point of view using Lachman and KT-1000/2000, and different results from other systematic revisions (Fig. 6) showed statistically significant differences with less anterior laxity in 45% of studies that included the DB technique^56,59^. Based on current evidence, DB reconstruction appears to have fewer re-ruptures and less antero-posterior and rotatory laxity. 4. Rotational stability (internal rotation and pivot shift) is slightly better but not significant in DB reconstruction. This benefit is especially important regarding the concept of reinjury since the persistence of rotational instability has become evident as a cause of rupture. Therefore, although both patients needed to maintain “anti-rotational” muscle enhancement, this should be more demanding in patients with the SB technique. These results present controversy from previous systematic reviews showing that DB provided significantly better knee stability than the SB technique^10,56,58,59^. According to results presented by our work, DB was better in the long-term follow-up than the SB technique, but not in all follow-ups of the included studies. Similar to a previous systematic review^58^, heterogeneity was present in some outcomes, but there was no evidence of publication bias for any outcome. The double-bundle technique may be better than the single-bundle ACL reconstruction technique for rotational stability but not for function, translation, and complications.

As previously shown in studies and meta-analysis, there is a biomechanical improvement in postoperative knee stability by using DB reconstruction technique^59–61^. However, in the rotational stability, only 2 studies have shown statistical results in favour of the DB^40,51^, compared to several studies that present better objective scores^46,49^.

The need for this systematic review and meta-analysis is based on the joint interpretation of mid- and long-term clinical outcomes of knee functionality, objective measurements of knee stability, rotational knee stability and knee anterior stability, sports reincorporation and subjective assessments to confirm the current discordant results in SB and DB ACL reconstruction.

### Limitations

Only studies published in English were included, and therefore, some relevant studies may have been missed. The “*anatomical technique*” that uses the anteromedial portal to perform femoral tunnels has not been included as a study variable as it is a relatively new technique and still without relevant RCTs to complete the meta-analysis proposed by the research team; there is a proposal for a future meta-analysis of the interventions with this new procedure. Associated complications that may influence the failure of the plasty and the assessment of future osteoarthritis were not assessed. The risk of bias was unclear, and heterogeneity was moderate to high in several outcomes.

## Conclusion

Currently, there are no clear or significant differences in clinical stability and knee function or in sports incorporation for the recommendation of DB in the primary ACL. The true difference between both techniques is shown in the subjective assessment by the IKDC score reported by patients.

## Methods

### Systematic search

We used the Preferred Reporting Items for Systematic Reviews and Meta-analyses (PRISMA) to conduct this systematic review and meta-analysis^62^ in conformity with updated guidance of The Cochrane Collaboration Principles for Systematic Reviews^63^. Inclusion criteria were (1) participants older than 14 years with a ruptured ACL isolated or combined with other soft tissue injuries requiring ACL reconstruction; (2) randomized clinical trial design; (3) comparison of a single-bundle with a double-bundle (3 t or 4 t); and (4) main outcome measures such as measurements of knee functionality as measured by the patient or by the doctor (i.e. subjective and objective International Knee Documentation Committee Knee Form (IKDC) score) and a return to pre-injury activity levels (Tegner activity score and Lysholm score). Secondary outcome measures: objective measurements of knee stability (rotational knee stability (pivot-shift test and range of mobility of internal rotational)) and knee anterior stability was measured using the Lachman test and KT-1000/2000; and (5) publication between database inception and July 2019.

We developed comprehensive search strategies with the assistance of a health sciences documentalist with background in searching for systematic reviews including both index and keyword methods for PubMed, Embase (Elsevier platform), the Cochrane Central Register of Controlled Trials (Wiley platform), Web of Science, and CINAHL (Cumulative Index to Nursing and Allied Health Literature; EBSCO platform). To maximize sensitivity, no pre-set limits for the database were used. The PubMed search strategy was adapted for use with the other electronic databases. Complete search strategies are shown in Table 4.

**Table 4:** Search strategy.

#26	Search ((anterior cruciate ligament) AND ((((((((joint instability) OR orthopedic procedures) OR tendon transfer) OR arthroscopy) OR reconstructive surgical procedures) OR transplants) OR autologous transplantation) OR autologous transplantation)) AND ((("double-bundle?" or "double bundle?" or anatomic?)) AND ("single-bundle?" or "single bundle?" or anatomic?))
#25	Search ((anterior cruciate ligament) AND ((((((((joint instability) OR orthopedic procedures) OR tendon transfer) OR arthroscopy) OR reconstructive surgical procedures) OR transplants) OR autologous transplantation) OR autologous transplantation)) AND ((("double-bundle?" or "double bundle?" or anatomic?)) AND ("single-bundle?" or "single bundle?" or anatomic?))
#24	Search (("double-bundle?" or "double bundle?" or anatomic?)) AND ("single-bundle?" or "single bundle?" or anatomic?)
#23	Search ((((((graft?) OR reconstruct) OR reconstruct?) OR autograft?) OR autoplasty) OR allograft?) OR homograft?
#22	Search homograft?
#21	Search allograft?
#20	Search autoplasty
#19	Search autograft?
#18	Search reconstruct?
#17	Search reconstruct
#16	Search graft?
#15	Search (((((((joint instability) OR orthopedic procedures) OR tendon transfer) OR arthroscopy) OR reconstructive surgical procedures) OR transplants) OR autologous transplantation) OR autologous transplantation
#14	Search (((((((joint instability) OR orthopedic procedures) OR Tendon transfer) OR Arthroscopy) OR Reconstructive Surgical procedures) OR Transplants) OR Autologus transplantation) OR autologous transplantation
#13	Search "single-bundle?" or "single bundle?" or anatomic?
#12	Search "double-bundle?" or "double bundle?" or anatomic?
#11	Search "double-bundle" or "double bundle" or anatomic
#10	Search double-bundle$ or double bundle$ or anatomic$
#9	Search autologous transplantation
#8	Search Autologus transplantation
#7	Search Transplants
#6	Search Reconstructive Surgical procedures
#5	Search Arthroscopy
#4	Search Tendon transfer
#3	Search orthopedic procedures
#2	Search joint instability
#1	Search anterior cruciate ligament

The search was conducted in July 2019. In addition to the databases, we searched the reference lists of relevant studies and proceedings of orthopaedic conferences. The search results were exported to Excel (Microsoft Office 365 ProPlus) and duplicates were electronically removed.

Two investigators and one experienced senior orthopaedic surgeon independently screened records by title and abstract. In addition, records included by the first screening were assessed through a full-text review. Any discrepancies between the reviewers were resolved through discussion, and when necessary, a third reviewer was consulted.

### Data extraction

Two investigators individually extracted data from eligible studies using a data collection form. Discrepancies were resolved through discussion with a third reviewer. The following data elements were extracted: the name of the first author, publication year, design, patient characteristics, interventions (single-bundle and double bundle), outcomes (Lysholm score, Pivot shift, Range of mobility (degrees), Internal rotational range (mm), KT-1000/2000, Tegner score, Lachman test, Subjective and Objective IKDC score, statistical analyses, and results.

### Risk of bias assessment

The Cochrane Collaboration’s tool for assessing risk of bias (RoB) was used to evaluate the study risk of bias within the included randomized controlled trials^63^. Three investigators independently extracted information on randomization, allocation concealment, blinding, attrition, selective reporting, and other biases (manufacturer funding and statistical power) for each study. After discussion, categories for all included studies were graded as having a low, unclear, or high risk of bias.

### Statistical analysis

We used Review Manager software (RevMan, version 5.3; The Cochrane Collaboration, Copenhagen, Denmark) to perform statistical analyses. Heterogeneity was evaluated using the I^2^ calculation. I^2^ values were interpreted using the Cochrane criteria for measuring heterogeneity^64^. We used random-effects models for studies and although similar to the surgical technique, they showed high heterogeneity and fixed-effects for studies with low heterogeneity. We also calculated mean differences for continuous data (Lysholm score, range of mobility, internal rotational range, KT-1000/2000, Tegner score and objective IKDC score) and pooled risk differences (RD) for dichotomous data (pivot shift, Lachman test and subjective IKDC score) with 95% confidence intervals (CIs); *P* ≤ 0.05 was considered statistically significant.

## Data Availability

The datasets generated during and/or analysed during the current study are available from the corresponding author on reasonable request.
